# CLC-Pred: A freely available web-service for *in silico* prediction of human cell line cytotoxicity for drug-like compounds

**DOI:** 10.1371/journal.pone.0191838

**Published:** 2018-01-25

**Authors:** Alexey A. Lagunin, Varvara I. Dubovskaja, Anastasia V. Rudik, Pavel V. Pogodin, Dmitry S. Druzhilovskiy, Tatyana A. Gloriozova, Dmitry A. Filimonov, Narahari G. Sastry, Vladimir V. Poroikov

**Affiliations:** 1 Department for Bioinformatics, Institute of Biomedical Chemistry, Moscow, Russia; 2 Department for Bioinformatics, Medico-Biologic Faculty, Pirogov Russian National Research Medical University, Moscow, Russia; 3 Centre for Molecular Modeling, CSIR-Indian Institute of Chemical Technology, Hyderabad, India; Wayne State University, UNITED STATES

## Abstract

*In silico* methods of phenotypic screening are necessary to reduce the time and cost of the experimental *in vivo* screening of anticancer agents through dozens of millions of natural and synthetic chemical compounds. We used the previously developed PASS (Prediction of Activity Spectra for Substances) algorithm to create and validate the classification SAR models for predicting the cytotoxicity of chemicals against different types of human cell lines using ChEMBL experimental data. A training set from 59,882 structures of compounds was created based on the experimental data (IG50, IC50, and % inhibition values) from ChEMBL. The average accuracy of prediction (AUC) calculated by leave-one-out and a 20-fold cross-validation procedure during the training was 0.930 and 0.927 for 278 cancer cell lines, respectively, and 0.948 and 0.947 for cytotoxicity prediction for 27 normal cell lines, respectively. Using the given SAR models, we developed a freely available web-service for cell-line cytotoxicity profile prediction (CLC-Pred: Cell-Line Cytotoxicity Predictor) based on the following structural formula: http://way2drug.com/Cell-line/.

## Introduction

Oncology diseases are one of the main causes of death in the world [1]. Despite the fact that the development of antineoplastic agents is a main area for the biggest pharmaceutical companies (http://spotfire.tibco.com/en/demos/pharma-pipeline-analysis.aspx), the complexity of tumours and their histological, morphological and genetic diversity require the creation of new potent and safe drugs. Notwithstanding the progress in cell-based screening technology, experimental *in vivo* screening of anticancer drug-candidates through dozens of millions of natural and synthetic chemical compounds is rather expensive and time-consuming [2]. Different *in vitro* and *in silico* tools were proposed to reduce the cost of such screening and to reveal possible mechanisms of the growth inhibition and killing of tumour cells [3]. The study of the cytotoxicity of chemicals against tumour cell lines is widespread in the early stages of drug development, drug repositioning and cancer research [4]. NCI60, a 60 human tumour cell line anticancer drug screening panel, is one of the most well-known assays developed by the National Cancer Institute in the late 1980s for anticancer drug screening to replace the use of transplantable animal tumours [5]. Although the NCI has screened anticancer compounds for dozens of years, only approximately 70,000 compounds were estimated [5]. Since then, hundreds of cancer cell lines covering tissues and the histological variety of tumours have been developed for anticancer screening. Thus, the Genomics of Drug Sensitivity in Cancer project provides the cytotoxicity data for 138 drugs tested on 714 cancer cell lines (October 2013) [6,7], and the Center for Molecular Therapeutics provides a panel with 1200 human cancer cell lines (CMT1000) [8]. Despite such progress, these tools are still rather expensive and are only used by a limited number of scientists for a limited number of chemicals. Therefore, there is a clear need for computer-based tools for virtual drug screening and an evaluation of the selective cytotoxic effect of chemical compounds on cancer cell lines. There are several approaches to developing such tools:

(Q)SAR approach—analysis of “structure-cytotoxicity” relationships [9–14];
Connectivity map approach—comparison of drug-induced gene signatures given on tumour cells [15–19];
Network pharmacology approach—analysis of known drug-target interactions [20–23];
Machine learning techniques for revealing relations between cell line cytotoxicity (IC50, IG50) and microarray data [24–27].

Despite the variety in the proposed methods and successful cases of their application, all of them require significant supplementary intellectual and technological efforts or additional experimental studies (e.g., in a case of the use of microarray data). The approaches related to the analysis of microarray data cannot be used for the virtual screening of compounds that have not been synthesized yet and that have no microarray data. Moreover, the mean accuracy (AUC) of the CMap approach validated on an independent set is approximately 0.61 for antineoplastic drugs [28]. The most published QSAR-based methods for the prediction of chemical tumour cell line cytotoxicity aimed to create QSAR models for calculating the IC50 or IG50 values for the single cell line [13], cell lines belonging to an appropriate tissue [11,12] or without the determination of the particular cell line [10]. Only Menden and co-authors created QSAR models for 608 tumour cell lines from different tissues. The coefficient of determination R2 for the predicted IC50 values was 0.72 and 0.64 in an 8-fold cross-validation and an independent blind test, respectively [9]. However, their models were limited in applicability domain because they were based only on the data for 111 drugs tasted in the framework of the Genomics of Drug Sensitivity in Cancer project [9]. It is the smallest part of the known experimental data on chemical cytotoxicity. Moreover, none of the authors that predicted cytotoxicity against cancer cell lines predicted the cytotoxicity against human normal cell lines.

The recent development of freely available recourses, such as ChEMBL [29] and PubChem [30], containing experimental data from the biological testing of chemicals (including the cytotoxicity data of chemicals against tumour and normal cell lines) gives us an opportunity to use these data as an estimation of cell line cytotoxicity for new chemicals. Because these data are highly heterogenic and were obtained in different experimental conditions, it is likely that only kNN and classification SAR modelling can be used. PASS (Prediction of Activity Spectra for Substances) is a software related to the classification QSAR methods for predicting the biological activity of chemicals based on their 2D structure, which has been in development by the authors for many years for its use in predicting the biological activity on different levels of hierarchy of biological systems, including the mechanisms of actions (interaction with targets) and general effects (e.g., anti-inflammatory action, antihypertensive, antiepileptic, nootropic effects) [31–35]. We previously showed the applicability of PASS for predicting the carcinogenicity of drug-like organic compounds [36,37]. Because the cell line cytotoxicity of chemicals may be considered a type of biological activity, we used PASS for predicting cell line cytotoxicity. The successful use of PASS in the prediction of cytotoxicity for 24 breast cancer cell-lines was earlier demonstrated, when 49 compounds from more than 1 million commercially available samples of compounds were selected on the basis of the prediction results of breast cancer cell line cytotoxicity and an interaction with potential antineoplastic drug targets [38]. Experimental testing of the 49 selected compounds revealed nine new compounds with cytotoxicity against the MDA-MB-231 and MCF7 breast cancer cell lines with the IC50 value equal to 0.8 μM at the most active compound [38]. The aim of the current study was to create and validate the SAR models for predicting the cytotoxicity of chemicals against tumour and normal human cell lines belonging to different tissues based on the PASS approach and the ChEMBL cytotoxicity data and to implement these SAR models as a freely available web-service.

## Materials and methods

### PASS (Prediction of Activity Spectra for Substances) approach

PASS provides simultaneous predictions for many types of biological activities (activity spectrum) based on the structure of drug-like compounds [31–35]. In PASS, biological activities are described qualitatively (active or inactive). The activity spectrum of a chemical compound is the set of different biological activity types that reflect the results of the compound’s interaction with various biological entities. We consider that the cell line cytotoxicity of compounds is a biological activity because this effect is a response to the drug action and relates to the drug’s structure. The algorithm of the activity spectrum estimation is based on the naive Bayes approach with some significant enhancements [31,32].

The molecular structure is represented by the set of unique sub-structural atom-centric Multilevel Neighbourhoods of Atoms (MNA) descriptors of the first and second levels. These descriptors are a linear notation of atom-centred fragments in the structure of an organic molecule [39]. They are based on the molecular structure representation, which includes the hydrogen atoms according to the valences and partial charges of the atoms and does not specify the types of bonds. An example of the structural presentation by MNA descriptors for Sorafenib (used for the treatment of renal cell and hepatocellular carcinomas) is shown in S1 Fig of Supplements. The MNA descriptors are generated and prediction is executed only if the molecule’s structure corresponds to the following criteria:

- Each atom in a molecule must be presented by an atomic symbol from the periodic table. Symbols of unspecified atom A, Q, *, or R group labels are not allowed.
- Each bond in a molecule must be a covalent bond represented by single, double or triple bond types only.
- The structure must include three or more carbon atoms.
- The structure must include only one component.
- The structure must be neutralized.
- The absolute molecular weight of a compound must be less than 1250.

Since MNA descriptors do not represent the stereochemical peculiarities of the molecule, the substances with stereochemically different structures are formally considered to be equivalent.

A leave-one-out cross-validation for all predictable types of biological activity and all substances in the PASS training set provides an estimate of the PASS prediction accuracy during the training procedure. The accuracy criterion ROC AUC (the Area Under the ROC Curve) is used. It is the estimate of the probability that positive and negative examples (active and inactive compounds) that are arbitrarily chosen from a validation set may be classified correctly by the prediction.

The predicted activity spectrum in PASS is represented by a list of activities with probabilities «to be active» ***Pa*** and «to be inactive» ***Pi***. The list of predicted activities is arranged in descending order according to ***Pa****–****Pi*** values. Thus, the more probable activity types are at the top of the list. If the user chooses a higher value of ***Pa*** as a cut-off for selection of probable activities, the chance to confirm the predicted activities by the experiment is also high, but many existing activities will be lost. For instance, if ***Pa***>0.5 is used as a threshold, about half of the real activities will be lost; for ***Pa***>0.7, the portion of lost activities is 70%, etc.

By definition, the probabilities ***Pa*** and ***Pi*** are measures that belong to both subsets of "active" and "inactive" compounds and the probabilities of the 1^st^ and 2^nd^ types of prediction error, respectively. These two interpretations of probabilities ***Pa*** and ***Pi*** are equivalent and can be used for interpreting the results of prediction. They can also be used for the construction of different criteria to predict the results of the analysis corresponding to the specific practical tasks.

### Training dataset

The ChEMBL database was used as a resource for cytotoxicity data of chemicals [29]. The database was chosen because of its convenience, free access, standardization and curation of the data. The twenty third version of the ChEMBL (ChEMBL_23) loaded into the MySQL database (http://dev.mysql.com/) was used. The script for the generation of the training sets was written in PHP language. ChEMBL_23 contained data for more than 1.7 million compounds, with information regarding their structures and interactions with over 11.5 thousand targets, including human tumour and normal cell lines. Two training sets were created from the ChEMBL data. One of the training sets contained the data on chemical cytotoxicity against human tumour cell lines, and the one was for human normal cell lines. The names of the cell-lines were used as in ChEMBL to provide links to the experimental data. The data from ChEMBL and Cellosaurus were used to distinguish cancer cell lines from non-cancer ones. Database of Cross-Contaminated or Misidentified Cell Lines was used to find in ChEMBL and to exclude from our training set misidentified cell lines where no authentic stock was ever found [40].

Structure Data File (SDF) format was used to save the extracted information. Single small molecular-weight organic compounds with electroneutral structures were selected during the creation of the training sets. The IG50 (half maximal inhibitory growth), IC50 (half maximal inhibitory concentration) and % inhibition (of activity) values were analysed. The compounds were considered active if the IG50 and IC50 values were less than 10000 nM or if the percent of inhibition was higher than 50%. All compounds were considered inactive for the appropriate cell line if they were not active for this cell line according to the above-mentioned criteria. The selected cell lines contained at least 3 active and 10 inactive compounds. All the records of compounds that were simultaneously classified as active and inactive for the appropriate cell line were excluded.

## Results

### Creation and validation of SAR models for the prediction of cell line cytotoxicity

The training set of 59,882 unique structures of compounds was created based on the experimental data from the ChEMBL (version 23), which reflects the current knowledge about the cytotoxic substances to 943 human cell lines. This training set was used to train PASS for the creation of classification models of “structure-cytotoxicity” relationships. Only cell lines for which the cytotoxicity was predicted with the accuracy of prediction (AUC) higher than 0.8 were selected. The average accuracy of prediction calculated by a leave-one-out cross-validation (LOO CV) procedure was 0.948 for the cytotoxicity prediction for 27 normal cell lines (Table 1) and was 0.930 for 278 cancer cell lines (S1 Table). The average accuracy of prediction calculated by a 20-fold cross-validation procedure was similar to the result given for the LOO CV: 0.947 for cytotoxicity prediction for 27 normal cell lines (Table 1) and 0.927 for 278 cancer cell lines (S1 Table). The small difference between the accuracy of prediction given by the LOO CV and the 20-fold CV procedures shows the creation of robust SAR models.

**Table 1:** Normal cell lines with predicted accuracy calculated by leave-one-out cross-validation (AUC LOO CV) and 20-fold cross-validation (AUC 20-fold CV) procedures.

No	Cell line	Type of cell line	Tissue/organ	N	AUC LOO CV	AUC 20-fold CV
1	AG1523	Fibroblast	Fibroblast	25	0.971	0.971
2	BJ	Foreskin fibroblast	Foreskin	37	0.889	0.862
3	CRL-7065	Fibroblast	Skin	9	0.926	0.927
4	Detroit 551	Embryonic skin	Skin	30	0.962	0.962
5	HaCaT	Keratinocyte	Skin	218	0.978	0.978
6	HASMC	Aortic smooth muscle	Muscle	26	0.999	0.999
7	HEK293	Embryonic kidney fibroblast	Kidney	711	0.922	0.921
8	HEL 299	Fibroblast	Lung	3	0.889	0.891
9	HFF	Foreskin fibroblast	Skin	171	0.974	0.974
10	HFL1	Human foetal lung fibroblast	Lung	3	1.000	1.000
11	HMEC	Microvascular endothelial cell	Breast	64	0.948	0.950
12	HS27	Fibroblast	Skin	40	0.971	0.972
13	HUVEC	Umbilical vein endothelial cell	Endothelium	999	0.958	0.958
14	IMR-90	Embryonic lung fibroblast	Lung	14	0.860	0.862
15	MRC5	Embryonic lung fibroblast	Lung	392	0.921	0.920
16	MT2	Lymphocyte (HTLV-1 producing cell line)	Blood	93	0.968	0.969
17	NFF	Fibroblast	Skin	57	0.978	0.978
18	NHDF	Fibroblast	Skin	51	0.947	0.941
19	PBMC	Peripheral blood mononuclear cell	Blood	1194	0.973	0.972
20	PrEC	Prostate epithelial cell	Prostate	4	0.802	0.804
21	RPTEC	Renal proximal tubule epithelial cells	Kidney	8	0.998	0.998
22	SKW 6.4	B lymphocyte; Epstein-Barr virus (EBV) transformed	Haematopoietic, lymphoid tissue	39	1.000	1.000
23	TERT-RPE1	Retinal pigmented epithelial cell	Retina	10	0.903	0.904
24	WI-38	Embryonic lung fibroblast	Lung	150	0.939	0.939
25	WI-38 VA13	Embryonic lung fibroblast	Lung	6	0.965	0.965
26	WIL2	Lymphoblastoid cell	Haematopoietic, lymphoid tissue	31	1.000	1.000
27	WIL2-NS	Lymphoblastoid cell	Haematopoietic, lymphoid tissue	44	0.961	0.953
**Mean**					**0.948**	**0.947**

N—number of active compounds in the training set

Table 2 represents the cancer cell lines’ distribution in 27 various organs or tissue types. More than 10000 compounds were actives for the colon (18 423), breast (15 716) and lung (14 439) tumour cell lines, probably because many cell lines are known for these organs and they are the objects of intensive study.

**Table 2:** Distribution of cancer cell lines in various organs or tissue types with data on mean accuracy of prediction calculated by leave-one-out cross-validation (AUC LOO CV) and 20-fold cross-validation (AUC 20-fold CV) procedures for cell lines from the Organ/tissue.

No	Organ/tissue	Number of cell lines	N	AUC LOO CV	AUC 20-fold CV
1	Adrenal cortex	1	11	0.844	0.846
2	Blood	26	7232	0.950	0.950
3	Bone	8	443	0.902	0.901
4	Brain	15	3534	0.941	0.940
5	Breast	16	15716	0.915	0.914
6	Cervix	3	4425	0.935	0.934
7	Colon	26	18423	0.948	0.947
8	Germ cell, fibroblast	1	58	0.993	0.992
9	Haematopoietic and lymphoid tissue	16	9540	0.914	0.912
10	Head and neck	4	82	0.989	0.989
11	Kidney	11	4678	0.904	0.902
12	Large intestine	1	564	0.962	0.961
13	Liver	8	3165	0.960	0.959
14	Lung	38	14439	0.915	0.911
15	Nervous system	3	599	0.920	0.921
16	Ovarium	24	7408	0.942	0.941
17	Pancreas	14	1417	0.921	0.919
18	Prostate	7	7286	0.935	0.933
19	Skin	26	7386	0.910	0.908
20	Small intestine	1	8	1.000	1.000
21	Soft tissue	1	338	0.903	0.899
22	Stomach	14	1884	0.948	0.948
23	Testicle	1	16	0.986	0.986
24	Thyroid	2	166	0.919	0.913
25	Upper aerodigestive tract	2	64	0.981	0.792
26	Urinary tract	6	583	0.913	0.908
27	Uterus	3	138	0.954	0.954

N—number of active compounds in the training set.

The AUC range for the different cell lines was from 0.800 (DMS-114—lung carcinoma) to 1.000 (e.g., MOH—cisplatin-resistant ovarian carcinoma cells). The most accurate prediction among organs with several cell lines was obtained for four head and neck cell lines (AUC 0.989; 82 active compounds in the training set), two upper aerodigestive tract cell lines (AUC 0.981; 64), and eight liver cell lines (AUC 0.960; 3165). The colon, breast, and lung cell lines also showed a mean accuracy of prediction of AUC > 0.9 (0.948, 0.915, 0.915, respectively), but no strict correlation was found between the number of substances in the training set or the number of cell lines in the category and accuracy of prediction.

### CLC-Pred (Cell Line Cytotoxicity Predictor) web service

**Fig 1:**
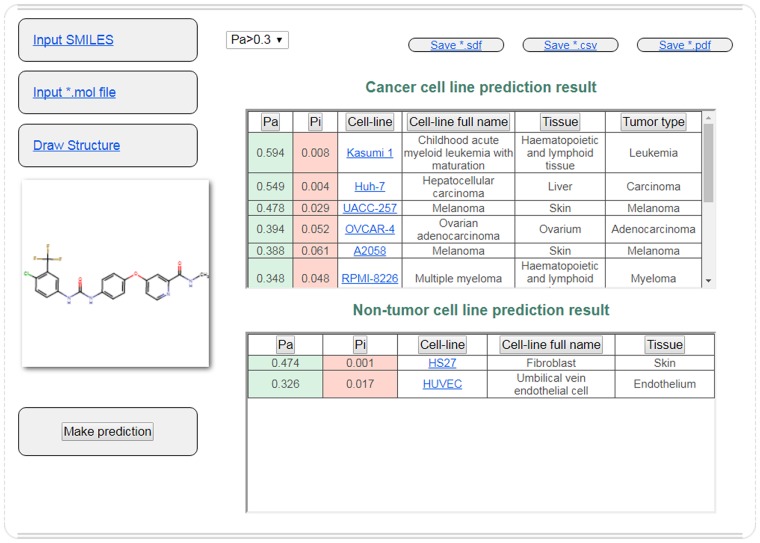
The prediction results for Sorafenib with the web-service.

A freely available web-service PASS CLC Pred for the prediction of cytotoxicity of chemicals against tumour and normal human cell lines from different tissues was created based on the abovementioned PASS models (http://www.way2drug.com/Cell-line/). The example of the prediction of cell line cytotoxicity for Sorafenib (a kinase inhibitor used for the treatment of liver, kidney and thyroid cancers) is shown in Fig 1. It displays that one of the top predicted tumour cell line is liver carcinoma, which coincides with its known therapeutic application. The prediction results also include the cytotoxicity against several melanoma cell lines, and some publications confirm this activity (e.g., Pécuchet with co-authors [41]).

The chemical structure for the prediction of cytotoxicity can be uploaded using the following three different modes: input SMILES strings [42], upload a file in a “mol file’ format [43] or draw in Marvin JS applet. The prediction results display two tables with the probable profiles of cytotoxicity (for tumour and normal cell lines). Each table includes the Pa and Pi values, the short and full names of cell lines, and the name of the tissue. The table with the prediction results against the tumour cell lines also includes the types of tumours. The prediction results can be sorted by clicking on the titles of the columns. The short names of cell lines have a link to a record from ChEMBL with the description of the cell line and experimental data of the compounds tested on this cell line. The prediction results can be saved as *.sdf, *.csv or *.pdf files.

The web-service uses a MySQL server to store data and PHP and HTML codes to implement the main interface. The Python script was used to produce the independent sub-processes for generating the input to the prediction program and data processing.

### Repositioning of drugs used for breast cancer treatment

Using the CLC-Pred service we analysed four drugs launched for the treatment of breast cancer and studied them for new therapeutic applications against other types of tumours. All information on the names, structures, stage of study and therapeutic applications was obtained from the Thomson Reuters (currently, Clarivate Analytics) Integrity Database (Table 3).

**Table 3:** Known and new predicted applications for drugs launched for the treatment of breast cancer.

Name	Known therapeutic groups of application	Phases of study	Possible new applications based on prediction of cancer cell line cytotoxicity
Doxorubicin	cancer: non-small cell lung*, breast*, brain*, liver*, head and neck	*launched*—breast cancer*	bone cancer, stomach cancer, kidney cancer, skin cancer, tumours of haematopoietic and lymphoid tissue
		*phase II*—head and neck cancer	
		*preclinical*—liver cancer* (hepatocellular carcinoma), hepatoblastoma*	
Gemcitabine	cancer: small cell lung*, prostate*, non-small cell lung*, lung*, cervical*, neurologic*, melanoma*, breast*, ovarian*, brain*, bladder*, digestive/gastrointestinal*, sarcoma*, colorectal*, renal*, head and neck, pancreatic*, endocrine, unspecified body location/system, female reproductive system; myeloid leukaemia*, lymphoma*, non-Hodgkin’s lymphoma	*launched*–cancer: lung* (non-small cell*), pancreas*, ovary*, breast*, lymphoma*, bladder*, biliary	osteosarcoma
		*phase III*—rhinopharyngeal cancer, medac cholangiocarcinoma, leiomyosarcoma	
		*phase II/III*—head and neck cancer	
		*phase II*—fallopian tube cancer, Hodgkin’s lymphoma, T-cell lymphoma*, peripheral	
		*phase I*—bladder cancer* (urothelial carcinoma, transitional cell)	
Raloxifene	cancer: breast*, prostate	*launched*—breast cancer*	acute T-lymphoblastic leukemia
		*phase I—*prostate cancer (adenocarcinoma)	
Vinorelbine	cancer: multiple myeloma*, prostate*, non-small cell lung*, neurologic*, breast*; solid tumours*, non-Hodgkin’s lymphoma	*launched*—cancer: breast*, lung* (non-small cell lung carcinoma*)	small cell lung carcinoma, colon carcinoma, osteosarcoma, childhood acute myeloid leukaemia with maturation
		*phase II*–glioma	

*italic font*–phase of drug development;

*–correct prediction by CLC-Pred web-service.

The prediction results, which are shown in the Supplements (S2 Table), were compared with known and newly studied therapeutic applications of these drugs. The sign ‘*’ shows that the prediction results of the drug include the predicted cell line cytotoxicity related to this application. For most of the known applications, the appropriate cell lines were predicted correctly. The last column displays new applications for these drugs given by the prediction of the cancer cell line cytotoxicity.

## Discussion

The ChEMBL database provides freely available experimental data on the cytotoxicity study of compounds against more than 1500 cell lines. These data allowed us to create reasonable classification SAR models for predicting the cytotoxicity of chemicals against 305 cell lines. Most of these cell lines (278) are different types of tumours related to the organs or tissue, which are of great interest in the development of new drugs or the repositioning of known drugs. ChEMBL includes much less information about the cytotoxicity of compounds against normal cell lines. Therefore, the number of classification models for the prediction of cytotoxicity of chemicals against normal cell lines is less. The models were created only for 27 normal cell-lines, which also belong to different organs or tissues. The ability to predict the cytotoxic action of compounds against normal cell lines is very important for estimating the safety of drug-candidates because many of the cytotoxic compounds that are used against tumour cell lines are often cytotoxic for normal cells and must be excluded from drug-candidates. The prediction of the cytotoxicity of chemicals against normal cell lines may be helpful for estimating the safety of drug-candidates for development in other therapeutic fields of application. The estimation of the general toxicity of compounds at the level of the organism may be given by our previously developed freely available web service for the prediction of rat acute toxicity (http://www.way2drug.com/gusar/acutoxpredict.html), which predicts the LD50 values for chemicals with oral, intravenous, intraperitoneal and subcutaneous routes of administration [44]. The PASS Online (http://www.way2drug.com/PASSOnline/) service may be used for predicting the possible molecular mechanisms of action related to the cytotoxic action of compounds [34].

During the analysis of the prediction results provided by the CLC-Pred service, one should remember that the prediction of cytotoxicity against each cell line is executed independently from other cell lines with a different accuracy of prediction. With less AUC accuracy calculated by the LOO CV, a greater number of false positive results will be in the prediction results for a given cell line. If one would like to reduce the number of false positive predictions, he (she) should increase the threshold of the Pa value. Together with the rules of interpretation of the prediction results described in the materials and methods, it should be noted that prediction results including the cytotoxicity against several cell lines from the same organ or tissue increase the probability of finding cytotoxic compounds acting on tumours located in that organ or tissue. The web service started working in 2016. Since then, several studies to assess the cytotoxicity of natural compounds have been conducted by independent researchers [45–48].

The traditional single target or multi-target based drug designs aim at estimating the interaction between the ligand and target(s). In this paradigm, some potential drug-candidates that display *in silico* or *in vitro* interactions with antineoplastic targets may not reveal the general cytotoxic effect on cell lines. Revealing the cell effects depends not only on the interaction with the targets but also on many other parameters, e.g., interaction with transporters, passage through a cell membrane, and regulation of signal and metabolic pathways, which depend on cell-line-specific cancer mutations and changes in gene expression. Therefore, the combination of the computational estimation of the cytotoxic effect of chemicals in different cell-lines together with the estimation of the ligand-target interactions provides a more effective method for the design of new antineoplastic drugs. An example of the application of such approach was recently provided in our publication on the search for new compounds with cytotoxicity against breast cancer cell lines [38].

## Supporting information

S1 Fig Sorafenib’s molecular structure and its presentation by MNA descriptors. (TIF)

S1 Table Tumour cell lines with predicted accuracy calculated by leave-one-out cross-validation (AUC LOO CV) and 20-fold cross-validation (AUC 20-fold CV) procedures. (PDF)

S2 Table CLC-Pred prediction results for four drugs launched for treatment of breast cancer and studied for new therapeutic applications against other types of tumours. (PDF)

S1 File The script for the generation of SDF files of the training sets. (PHP)

S2 File The input file for Script.php with ChEMBL IDs and names of tumour cell lines for creation of SDF file of the training set. (CSV)

S3 File The input file for Script.php with ChEMBL IDs and names of non-tumour cell lines for creation of SDF file of the training set. (CSV)

## References

[1] Siegel R, Miller KD, Jemal A. Cancer statistics, 2015. CA Cancer J. Clin. 2015;65(1):5–29. doi: 10.3322/caac.21254 25559415 10.3322/caac.21254

[2] Chabner BA and Roberts TG Jr. Timeline—chemotherapy and the war on cancer. Nat Rev. Cancer. 2005;5:65–72. doi: 10.1038/nrc1529 15630416 10.1038/nrc1529

[3] Kim HS, Sung YJ, Paik S. Cancer Cell Line Panels Empower Genomics-Based Discovery of Precision Cancer Medicine. Yonsei Med. J., 2015;56(5):1186–1198. doi: 10.3349/ymj.2015.56.5.1186 26256959 10.3349/ymj.2015.56.5.1186 PMC4541646

[4] Li J, Zhao W, Akbani R, Liu W, Ju Z, Ling S, et al Characterization of Human Cancer Cell Lines by Reverse-phase Protein Arrays. Cancer Cell. 2017;31(2):225–239. doi: 10.1016/j.ccell.2017.01.005 28196595 10.1016/j.ccell.2017.01.005 PMC5501076

[5] Shoemaker RH. The NCI60 human tumour cell line anticancer drug screen. Nat Rev Cancer, 2006;6(10):813–823. doi: 10.1038/nrc1951 16990858 10.1038/nrc1951

[6] Garnett MJ, Edelman EJ, Heidorn SJ, Greenman CD, Dastur A, Lau KW, et al Systematic identification of genomic markers of drug sensitivity in cancer cells. Nature. 2012;483(7391):570–575. doi: 10.1038/nature11005 22460902 10.1038/nature11005 PMC3349233

[7] Yang W, Soares J, Greninger P, Edelman EJ, Lightfoot H, Forbes S., et al Genomics of Drug Sensitivity in Cancer (GDSC): a resource for therapeutic biomarker discovery in cancer cells. Nucleic Acids Res. 2013;41(Database issue):D955–D961. doi: 10.1093/nar/gks1111 23180760 10.1093/nar/gks1111 PMC3531057

[8] Sharma SV, Haber DA, Settleman J. Cell line-based platforms to evaluate the therapeutic efficacy of candidate anticancer agents. Nat Rev Cancer. 2010;10(4):241–253. doi: 10.1038/nrc2820 20300105 10.1038/nrc2820

[9] Menden MP, Iorio F, Garnett M, McDermott U, Benes CH, Ballester PJ, et al Machine learning prediction of cancer cell sensitivity to drugs based on genomic and chemical properties. PLoS One. 2013;8(4):e61318 doi: 10.1371/journal.pone.0061318 23646105 10.1371/journal.pone.0061318 PMC3640019

[10] Li GH and Huang JF. CDRUG: a web server for predicting anticancer activity of chemical compounds. Bioinformatics, 2012;28(24):3334–3335. doi: 10.1093/bioinformatics/bts625 23080119 10.1093/bioinformatics/bts625

[11] Speck-Planche A, Kleandrova VV, Luan F, Cordeiro MN. Rational drug design for anti-cancer chemotherapy: multi-target QSAR models for the in silico discovery of anti-colorectal cancer agents. Bioorg Med Chem. 2012;20(15):4848–4855. doi: 10.1016/j.bmc.2012.05.071 22750007 10.1016/j.bmc.2012.05.071

[12] Bonnet M, Flanagan JU, Chan DA, Lai EW, Nguyen P, Giaccia AJ, et al SAR studies of 4-pyridyl heterocyclic anilines that selectively induce autophagic cell death in von Hippel-Lindau-deficient renal cell carcinoma cells. Bioorg. Med. Chem. 2008;19(11):3347–3356. 10.1016/j.bmc.2011.04.042 PMC3115526 21561782

[13] Boik JC, and Newman RA. Structure-activity models of oral clearance, cytotoxicity, and LD50: a screen for promising anticancer compounds. BMC Pharmacol. 2008;8:12 doi: 10.1186/1471-2210-8-12 18554402 10.1186/1471-2210-8-12 PMC2442056

[14] Lee CL, Lin YT, Chang FR, Chen GY, Backlund A, Yang JC, et al Synthesis and biological evaluation of phenanthrenes as cytotoxic agents with pharmacophore modeling and ChemGPS-NP prediction as topo II inhibitors. PLoS One. 2012;7(5):e37897 doi: 10.1371/journal.pone.0037897 22666407 10.1371/journal.pone.0037897 PMC3362575

[15] Lamb J. The Connectivity Map: a new tool for biomedical research. Nat Rev Cancer., 2007;7(1):54–60. doi: 10.1038/nrc2044 17186018 10.1038/nrc2044

[16] Lamb J, Crawford ED, Peck D, Modell JW, Blat IC, Wrobel MJ, et al The Connectivity Map: using gene-expression signatures to connect small molecules, genes, and disease. Science. 2006;313:1929–1935. doi: 10.1126/science.1132939 17008526 10.1126/science.1132939

[17] Qu XA and Rajpal DK. Applications of Connectivity Map in drug discovery and development. Drug Discov Today. 2012;17(23–24):1289–1298. doi: 10.1016/j.drudis.2012.07.017 22889966 10.1016/j.drudis.2012.07.017

[18] McArt DG and Zhang SD. Identification of Candidate Small-Molecule Therapeutics to Cancer by Gene-Signature Perturbation in Connectivity Mapping. PLoS One. 2011;6(1):e16382 doi: 10.1371/journal.pone.0016382 21305029 10.1371/journal.pone.0016382 PMC3031567

[19] Karube K, Tsuzuki S, Yoshida N, Arita K, Kato H, Katayama M, et al Comprehensive gene expression profiles of NK cell neoplasms identify vorinostat as an effective drug candidate. Cancer Lett. 2013;333(1):47–55. doi: 10.1016/j.canlet.2012.12.022 23348693 10.1016/j.canlet.2012.12.022

[20] Azmi AS. Adopting network pharmacology for cancer drug discovery. Curr. Drug Discov. Technol. 2013;10(2):95–105. 23237672 10.2174/1570163811310020002

[21] Aguda BD. Network pharmacology of glioblastoma. Curr. Drug Discov. Technol. 2013;10(2):125–138. 23237675 10.2174/1570163811310020005

[22] Li L, Zhou X, Ching WK, Wang P. Predicting enzyme targets for cancer drugs by profiling human metabolic reactions in NCI-60 cell lines. BMC Bioinformatics, 2010;11:501 20932284 10.1186/1471-2105-11-501 PMC2964682

[23] Iorio F, Bosotti R, Scacheri E, Belcastro V, Mithbaokar P, Ferriero R, et al Discovery of drug mode of action and drug repositioning from transcriptional responses. Proc Natl Acad Sci U S A., 2010;107(33):14621–14626. doi: 10.1073/pnas.1000138107 20679242 10.1073/pnas.1000138107 PMC2930479

[24] Bayer I, Groth P, Schneckener S. Prediction errors in learning drug response from gene expression data—influence of labeling, sample size, and machine learning algorithm. PLoS One. 2013;8(7):e70294 doi: 10.1371/journal.pone.0070294 23894636 10.1371/journal.pone.0070294 PMC3720898

[25] Wan P, Li Q, Larsen JE, Eklund AC, Parlesak A, Rigina O, et al Prediction of drug efficacy for cancer treatment based on comparative analysis of chemosensitivity and gene expression data. Bioorg Med Chem. 2012;20(1):167–176. doi: 10.1016/j.bmc.2011.11.019 22154557 10.1016/j.bmc.2011.11.019

[26] Kim N, He N, Kim C, Zhang F, Lu Y, Yu Q, et al Systematic analysis of genotype-specific drug responses in cancer. Int. J. Cancer. 2012;131(10):2456–2464. doi: 10.1002/ijc.27529 22422301 10.1002/ijc.27529 PMC4012336

[27] Covell DG, Wallqvist A, Huang R, Thanki N, Rabow AA, Lu XJ. Linking tumor cell cytotoxicity to mechanism of drug action: an integrated analysis of gene expression, small-molecule screening and structural databases. Proteins. 2005;59(3):403–433. doi: 10.1002/prot.20392 15778971 10.1002/prot.20392

[28] Cheng J, Yang L, Kumar V, Agarwal P. Systematic evaluation of connectivity map for disease indications. Genome Med. 2014;6(12):540 doi: 10.1186/s13073-014-0095-1 25606058 10.1186/s13073-014-0095-1 PMC4278345

[29] Gaulton A, Hersey A, Nowotka M, Bento AP, Chambers J, Mendez D, et al The ChEMBL database in 2017. Nucleic Acids Res. 2017;45(D1):D945–D954. doi: 10.1093/nar/gkw1074 27899562 10.1093/nar/gkw1074 PMC5210557

[30] Wang Y, Bryant SH, Cheng T, Wang J, Gindulyte A, Shoemaker BA, et al PubChem BioAssay: 2017 update. Nucleic Acids Res. 2017;45(D1):D955–D963. doi: 10.1093/nar/gkw1118 27899599 10.1093/nar/gkw1118 PMC5210581

[31] Poroikov VV, Filimonov DA, Borodina YV, Lagunin AA, Kos A. Robustness of biological activity spectra predicting by computer program PASS for non-congeneric sets of chemical compounds. J. Chem. Inf. Comput. Sci. 2000;40(6):1349–1355. 11128093 10.1021/ci000383k

[32] Filimonov D and Poroikov V. Probabilistic approach in activity prediction In: Varnek A, Tropsha A, editors. Chemoinformatics Approaches to Virtual Screening. Cambridge, RSC Publishing, 2008, pp. 182–216.

[33] Lagunin A, Filimonov D, Poroikov V. Multi-Targeted Natural Products Evaluation Based on Biological Activity Prediction with PASS. Curr. Pharm. Des. 2010;16:1703–1717. 20222853 10.2174/138161210791164063

[34] Filimonov DA, Lagunin AA, Gloriozova TA, Rudik AV, Druzhilovskii DS, Pogodin PV, et al Prediction of the biological activity spectra of organic compounds using the PASS online web resource. Chem Heterocycl Compd. 2014;50:444–457.

[35] Murtazalieva KA, Druzhilovskiy DS, Goel RK, Sastry GN, Poroikov VV. How good are publicly available web services that predict bioactivity profiles for drug repurposing? SAR and QSAR in Environmental Research, 2017;28(10):843–862. doi: 10.1080/1062936X.2017.1399448 29183230 10.1080/1062936X.2017.1399448

[36] Lagunin AA, Dearden JC, Filimonov DA, Poroikov VV. Computer-aided rodent carcinogenicity prediction. Mutat. Res. 2005;586:138–146. doi: 10.1016/j.mrgentox.2005.06.005 16112600 10.1016/j.mrgentox.2005.06.005

[37] Lagunin A, Filimonov D, Zakharov A, Xie W, Huang Y, Zhu F, et al Computer-Aided Prediction of Rodent Carcinogenicity by PASS and CISOC-PSCT. QSAR Comb. Sci., 2009;28(8):806–810.

[38] Konova V, Lagunin A, Pogodin P, Kolotova E, Shtil A, Poroikov V. Virtual screening of chemical compounds active against breast cancer cell lines based on cell cycle modelling, prediction of cytotoxicity and interaction with targets. SAR QSAR Environ Res. 2015;26(7–9):595–604. doi: 10.1080/1062936X.2015.1076516 26358808 10.1080/1062936X.2015.1076516

[39] Filimonov D, Poroikov V, Borodina Yu, Gloriozova T. Chemical Similarity Assessment Through Multilevel Neighborhoods of Atoms: Definition and Comparison with the Other Descriptors. J. Chem. Inf. Comput. Sci. 1999;39(4):666–670.

[40] Capes-Davis A, Theodosopoulos G, Atkin I, Drexler HG, Kohara A, Macleod RA, et al Check your cultures! A list of cross-contaminated or misidentified cell lines. Int. J. Cancer. 2010;127(1):1–8. doi: 10.1002/ijc.25242 20143388 10.1002/ijc.25242

[41] Pécuchet N, Lebbe C, Mir O, Billemont B, Blanchet B, Franck N, et al Sorafenib in advanced melanoma: a critical role for pharmacokinetics? Br J Cancer. 2012;107(3):455–461. doi: 10.1038/bjc.2012.287 22767146 10.1038/bjc.2012.287 PMC3405224

[42] Weininger D. SMILES, a Chemical Language and Information System. 1. Introduction to Methodology and Encoding Rules. J. Chem. In.f Comput. Sci., 1988;28:31–36.

[43] Dalby A, Nourse JG, Hounshell WD, Gushurst AKI, Grier DL, Leland BA, et al Description of several chemical structure file formats used by computer programs developed at Molecular Design Limited. J. Chem. Inform. Comp. Sci. 1992;32:244–255.

[44] Lagunin A, Zakharov A., Filimonov D., Poroikov V. QSAR Modelling of Rat Acute Toxicity on the Basis of PASS Prediction. Mol. Inform. 2011;30(2–3):241–250. doi: 10.1002/minf.201000151 27466777 10.1002/minf.201000151

[45] Nand M, Maiti P, Pant R, Kumari M, Chandra S, Pande V. Virtual screening of natural compounds as inhibitors of EGFR 696–1022 T790M associated with non-small cell lung cancer. Bioinformation. 2016; 12(6):311–317. doi: 10.6026/97320630012311 28293073 10.6026/97320630012311 PMC5320927

[46] Maiti P, Nand M, Joshi H, Chandra S. Molecular docking analysis and screening of plant compounds against lung cancer target EGFR T790M mutant. Int J Comput Bioinfo In Silico Model. 2016; 5(2):787–792.

[47] Thomas A, Peter PKJ, Chandramohanakumar N. A Profiling of Anti-Tumour Potential of Sterols in the Mangrove Fern Acrostichum aureum. Int. J. Pharmacognosy and Phytochem. Res. 2016; 8(11); 1828–1832.

[48] Maiti P, Nand M, Kumari M, Pant R, Joshi H, Chandra S. Virtual Screening of EGFR Tyrosine Kinase Inhibitors Associated with Non-Small Cell Lung Cancer from Phytochemical Data Set. Journal of Emerging Trends in Computing and Information Sciences. 2016; 7(5):229–236.

