# Supplementary figures and images for: CLC-Pred: A freely available web-service for in silico prediction of human cell line cytotoxicity for drug-like compounds
Source: PLoS One. 2018 Jan 25;13(1):e0191838. doi: 10.1371/journal.pone.0191838 (PMC5784992; doi:10.1371/journal.pone.0191838)

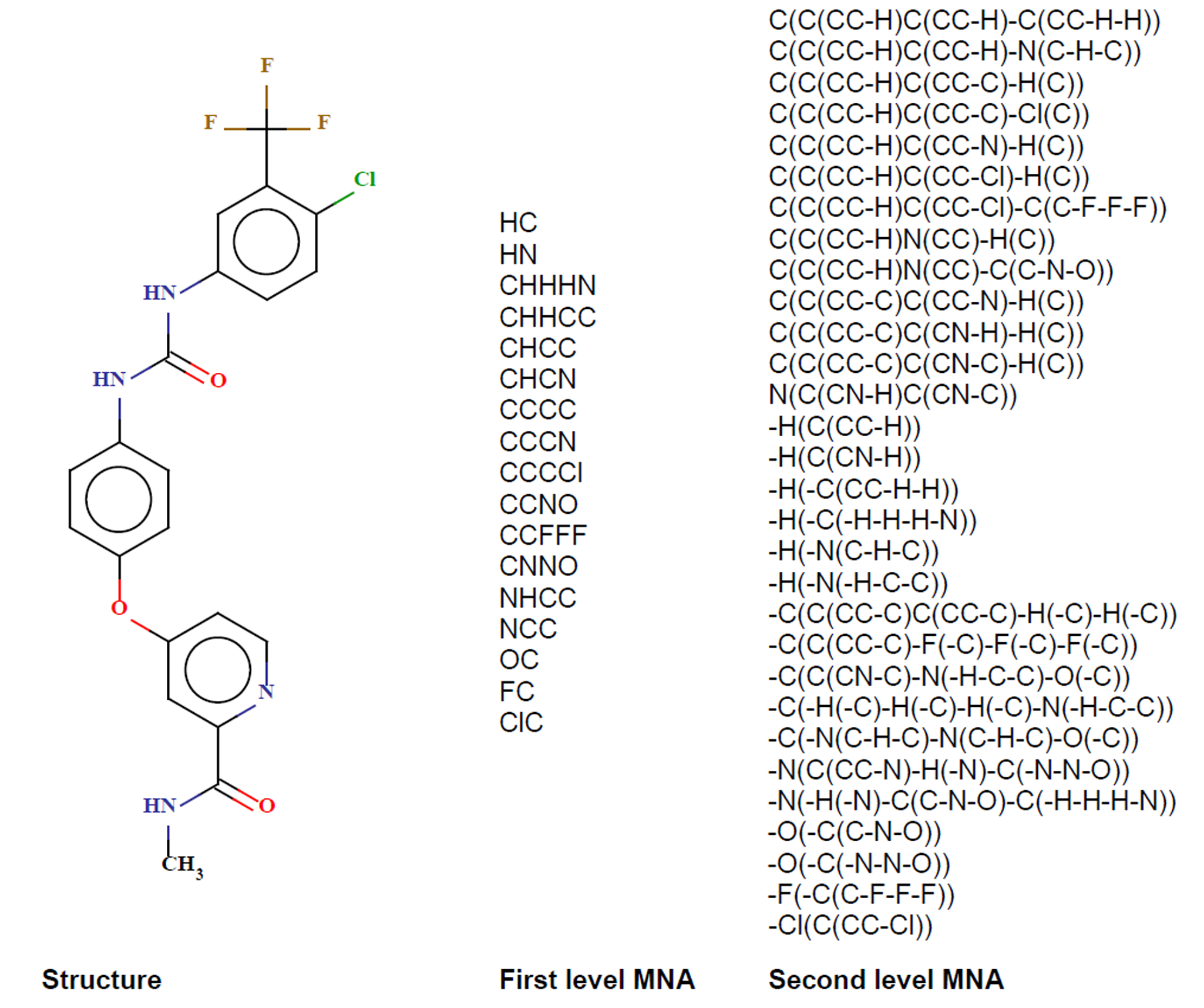

Supplement: S1 Fig — (TIF) [file pone.0191838.s001.tif]
