# Supplementary material for: CLC-Pred: A freely available web-service for in silico prediction of human cell line cytotoxicity for drug-like compounds
Source: PLoS One. 2018 Jan 25;13(1):e0191838. doi: 10.1371/journal.pone.0191838 (PMC5784992; doi:10.1371/journal.pone.0191838)
Supplement: S1 Table — (PDF) [file pone.0191838.s002.pdf]

S1 Table.

No	Cell line	Type of cell line	Organ/ Tissue	N	AUC LOO CV	AUC 20-fold CV
1	5637	Urothelial bladder carcinoma	Urinary tract	157	0.849	0.836
2	1A9	Ovarian adenocarcinoma	Ovarium	85	0.988	0.988
3	1A9/ptx-10	Ovarian adenocarcinoma	Ovarium	36	0.990	0.991
4	1A9/ptx-22	Ovarian adenocarcinoma	Ovarium	24	0.993	0.993
5	786-0	Renal carcinoma	Kidney	593	0.871	0.865
6	833K	Testicular embryonal carcinoma	Testicle	16	0.986	0.986
7	8505C	Thyroid gland undifferentiated (anaplastic) carcinoma	Thyroid	116	0.843	0.831
8	A 172	Glioblastoma	Brain	54	0.998	0.998
9	A-375	Malignant melanoma	Skin	759	0.940	0.939
10	A-427	Lung carcinoma	Lung	202	0.871	0.864
11	A-431	Epidermoid carcinoma	Skin	790	0.928	0.926
12	A121	Ovarian carcinoma	Ovarium	80	1.000	1.000
13	A2058	Melanoma	Skin	80	0.822	0.806
14	A2780	Ovarian carcinoma	Ovarium	1964	0.934	0.933
15	A2780cisR	Cisplatin-resistant ovarian carcinoma	Ovarium	30	0.925	0.924
16	A498	Renal carcinoma	Kidney	595	0.876	0.877
17	A549	Lung carcinoma	Lung	6627	0.865	0.865
18	ACHN	Papillary renal carcinoma	Kidney	876	0.898	0.896
19	ADR5000	Childhood T acute lymphoblastic leukemia	Blood	3	0.921	0.923
20	AGS	Gastric adenocarcinoma	Stomach	228	0.873	0.866
21	ASPC1	Pancreatic ductal adenocarcinoma	Pancreas	94	0.959	0.959
22	Bcap37	Breast adenocarcinoma	Breast	39	0.955	0.956
23	BE	Colon adenocarcinoma	Colon	30	1.000	0.999
24	BEL-7404	Hepatocellular carcinoma	Liver	5	1.000	1.000
25	BE-NQ	Colon adenocarcinoma	Colon	15	0.999	0.999
26	BGC-823	Stomach adenocarcinoma	Stomach	219	0.949	0.949
27	BT-474	Breast ductal carcinoma	Breast	403	0.940	0.940
28	BT-549	Breast ductal carcinoma	Breast	444	0.870	0.871
29	BXPC-3	Pancreatic adenocarcinoma	Pancreas	274	0.950	0.948
30	Bel-7402	Hepatoma	Liver	533	0.941	0.940
31	C180-13S	Ovarian carcinoma	Ovarium	11	0.999	0.999
32	C8166	Leukemic T-cells	Blood	100	0.837	0.841
33	Caco-2	Colon adenocarcinoma	Colon	182	0.924	0.921
34	CAKI-1	Kidney carcinoma	Kidney	592	0.860	0.857
35	CCRF-CEM	Childhood T acute lymphoblastic leukemia	Blood	2643	0.911	0.911
36	CCRF-CEM/VCR-1000	T-cell leukaemia	Blood	62	0.990	0.991
37	CCRF-HSB-2	Childhood T acute lymphoblastic leukemia	Blood	19	0.963	0.964
38	CCRF-SB	Childhood T acute lymphoblastic leukemia	Blood	91	0.968	0.968
39	CEM-0	T-cell leukemia	Blood	28	0.999	0.999
40	CEM-SS	Childhood T acute lymphoblastic leukemia	Blood	124	0.984	0.984

41	CEM-c113	Childhood T acute lymphoblastic leukemia	Blood	4	1.000	1.000
42	CEM/C2	Camptothecin-resistant CEM	Blood	15	0.953	0.958
43	CFPAC-1	Pancreatic carcinoma	Pancreas	38	0.809	0.787
44	CH1	Ovarian carcinoma	Ovarium	130	0.985	0.984
45	COLO 205	Colon adenocarcinoma	Colon	1152	0.907	0.907
46	COLO 320	Colon adenocarcinoma	Colon	6	0.988	0.980
47	COLO 320DM	Colon adenocarcinoma	Colon	6	1.000	1.000
48	CPT30	Nasopharyngeal carcinoma	Head and neck	7	1.000	1.000
49	CWR22R	Prostate carcinoma epithelial cell line	Prostate	123	0.863	0.849
50	CAOV-3	High grade ovarian serous adenocarcinoma	Ovarium	38	0.943	0.944
51	Col2	Colon carcinoma	Colon	101	0.977	0.977
52	D54	Glioblastoma	Brain	4	0.995	0.995
53	DAN-G	Human pancreas adenocarcinoma cell line	Pancreas	48	0.956	0.957
54	DLD-1	Colon adenocarcinoma	Colon	499	0.950	0.949
55	DMS-114	Lung carcinoma	Lung	103	0.800	0.789
56	DO4	Melanoma	Skin	6	0.998	0.998
57	DU-145	Prostate carcinoma	Prostate	2797	0.896	0.895
58	EKVX	Non-small cell lung carcinoma	Lung	262	0.881	0.880
59	FaDu	Hypopharyngeal squamous carcinoma	Upper aerodigestive tract	60	0.962	0.962
60	G-361	Melanoma	Skin	165	0.887	0.880
61	GBM	Glioblastoma multiforme	Brain	11	0.999	0.999
62	GC3/Cl	Colorectal carcinoma	Colon	5	1.000	1.000
63	GC3/M	Colorectal carcinoma	Colon	15	1.000	1.000
64	GC3/MTK	Colorectal carcinoma	Colon	54	1.000	1.000
65	GHOST CXCR4	Osteosarcoma	Bone	3	1.000	1.000
66	GLC4	Small cell lung carcinoma	Lung	10	0.999	0.999
67	H2981	Lung carcinoma	Lung	11	0.984	0.985
68	H322	Lung carcinoma	Lung	6	1.000	1.000
69	H9	T-lymphoid	Haematopoietic and lymphoid tissue	117	0.872	0.867
70	HA22T	Hepatocellular carcinoma	Liver	19	0.999	0.999
71	HBL	Melanoma	Skin	10	0.937	0.935
72	HCC 2998	Colon adenocarcinoma	Colon	348	0.880	0.881
73	HCT-116	Colon carcinoma	Colon	6502	0.888	0.887
74	HCT-116/VM46	Colon carcinoma	Colon	78	1.000	1.000
75	HCT-15	Colon adenocarcinoma	Colon	1322	0.897	0.895
76	HCT-8	Ileocecal adenocarcinoma	Large intestine	564	0.962	0.961
77	HeLa	Cervical adenocarcinoma	Cervix	4059	0.905	0.904
78	HepG2	Hepatoblastoma	Liver	2032	0.915	0.915
79	HEY	Ovarian carcinoma	Ovarium	29	0.987	0.987
80	HL-60	Promyeloblast leukemia	Haematopoietic and lymphoid tissue	3155	0.902	0.902
81	HN5	Squamous cell carcinoma	Skin	73	0.989	0.989
82	HNO 97	Oral cavity squamous cell carcinoma	Head and neck	12	0.999	0.999
83	HONE1	Nasopharyngeal carcinoma	Head and neck	30	0.999	0.999
84	HOP-18	Non-small cell lung carcinoma	Lung	9	0.913	0.915

85	HOP-62	Non-small cell lung carcinoma	Lung	867	0.894	0.893
86	HOP-92	Non-small cell lung carcinoma	Lung	413	0.843	0.836
87	HOS	Osteosarcoma	Bone	137	0.870	0.869
88	HOS-TE85	Osteosarcoma	Bone	13	0.859	0.860
89	HPAC	Pancreatic adenocarcinoma	Pancreas	21	0.979	0.979
90	HRT-18	Colon adenocarcinoma	Colon	4	1.000	1.000
91	Hs 683	Oligodendroglioma	Brain	4	0.879	0.883
92	Hs 766	Pancreatic carcinoma	Pancreas	9	1.000	1.000
93	Hs-578T	Invasive ductal breast carcinoma	Breast	152	0.876	0.876
94	HT-1080	Fibrosarcoma	Soft tissue	338	0.903	0.899
95	HT-29	Colon adenocarcinoma	Colon	4681	0.887	0.886
96	Huh-7	Hepatocellular carcinoma	Liver	120	0.928	0.927
97	HuP-T3	Pancreatic adenocarcinoma	Pancreas	61	0.870	0.850
98	HuT78	T-lymphoma	Haematopoietic and lymphoid tissue	28	0.971	0.972
99	HuTu80	Duodenal adenocarcinoma	Small intestine	8	1.000	1.000
100	Human lung LXF 629L tumor	Lung adenocarcinoma	Lung	7	0.981	0.981
101	IGROV-1	Ovarian adenocarcinoma	Ovarium	643	0.880	0.878
102	IMR-32	Neuroblastoma	Nervous system	81	0.965	0.965
103	Ishikawa	Endometrial adenocarcinoma	Uterus	45	0.974	0.974
104	J82	Bladder carcinoma	Urinary tract	167	0.879	0.871
105	JAM	Ovarian cystadenocarcinoma	Ovarium	5	0.998	0.998
106	Jurkat	Acute leukemic T-cells	Blood	1244	0.933	0.933
107	K562	Erythroleukemia	Haematopoietic and lymphoid tissue	3538	0.891	0.890
108	KARPAS-299	Anaplastic large cell lymphoma	Haematopoietic and lymphoid tissue	110	0.867	0.868
109	KATO III stomach cancer	Signet ring cell gastric adenocarcinoma	Stomach	23	0.991	0.991
110	Kasumi 1	Childhood acute myeloid leukemia with maturation	Haematopoietic and lymphoid tissue	63	0.814	0.814
111	KBM-3	Acute myelomonocytic leukemia	Blood	3	1.000	1.000
112	KBM-3/DOX	Acute myelomonocytic leukemia	Blood	3	1.000	1.000
113	KETR3	Renal carcinoma	Kidney	41	0.968	0.968
114	KM12	Colon adenocarcinoma	Colon	642	0.868	0.864
115	KKLS	Gastric adenocarcinoma	Stomach	4	0.999	0.999
116	L2987	Lung adenocarcinoma	Lung	11	0.972	0.972
117	LAPC4	Prostate carcinoma	Prostate	10	1.000	1.000
118	LNCaP	Prostate carcinoma	Prostate	918	0.952	0.953
119	LoVo	Colon adenocarcinoma	Colon	781	0.930	0.928
120	LOX IMVI	Melanoma	Skin	636	0.898	0.898
121	LS174T	Colon adencocarcinoma	Colon	27	0.864	0.864
122	Leukemia cells	Leukemia	Blood	8	0.889	0.890
123	Lu1	Lung carcinoma	Lung	57	0.954	0.953
124	Lung cancer cell line	Lung cancer	Lung	7	0.968	0.968
125	LXFL 529	Non-small cell lung carcinoma	Lung	14	0.963	0.965
126	M14	Melanoma	Skin	662	0.883	0.882

127	M19-MEL	Melanoma	Skin	9	0.956	0.957
128	M21	Melanoma	Skin	187	0.983	0.983
129	Malme-3M	Melanoma	Skin	403	0.875	0.875
130	Manca	Burkitt's line -high grade lymphoma	Haematopoietic and lymphoid tissue	8	1.000	1.000
131	MAXF401	Breast carcinoma	Breast	7	0.918	0.919
132	MCF7	Breast carcinoma	Breast	8126	0.866	0.865
133	MCF7R	Breast carcinoma	Breast	69	0.996	0.996
134	MCF7S	Breast carcinoma	Breast	38	0.971	0.947
135	MDA-MB-231	Breast adenocarcinoma	Breast	2999	0.891	0.890
136	MDA-MB-361	Breast adenocarcinoma	Breast	160	0.884	0.880
137	MDA-MB-453	Breast adenocarcinoma	Breast	70	0.802	0.810
138	MDA-MB-468	Breast adenocarcinoma	Breast	656	0.892	0.892
139	MES-SA	Uterine corpus sarcoma	Uterus	48	0.964	0.964
140	MES-SA/DXS	Uterine corpus sarcoma	Uterus	45	0.923	0.923
141	MEXF276L	Xenograft melanoma	Skin	4	0.807	0.808
142	MEXF989	Xenograft melanoma	Skin	4	0.807	0.808
143	MIA PaCa-2	Pancreatic carcinoma	Pancreas	504	0.912	0.909
144	MKN-45	Gastric adenocarcinoma	Stomach	556	0.961	0.961
145	MKN-7	Gastric carcinoma	Stomach	33	0.859	0.861
146	MM96L	Melanoma	Skin	62	1.000	1.000
147	MOH	Human B lymphocyte. EBV transformed	Blood	5	1.000	1.000
148	MOLT-3	T-lymphoblastic leukemia	Blood	85	0.982	0.980
149	MOLT-4	Acute T-lymphoblastic leukemia	Blood	946	0.874	0.873
150	MOVP-3	Adult T acute lymphoblastic leukemia	Blood	3	1.000	1.000
151	MT4	Adult T acute lymphoblastic leukemia	Blood	405	0.980	0.980
152	MV4-11	Myeloid leukemia	Haematopoietic and lymphoid tissue	356	0.972	0.973
153	MX1	Breast carcinoma	Breast	215	0.978	0.978
154	MeWo	Melanoma	Skin	8	0.879	0.879
155	Melanoma cells	Melanoma	Skin	5	0.912	0.913
156	NALM-6	Adult B acute lymphoblastic leukemia	Haematopoietic and lymphoid tissue	111	0.812	0.801
157	NB-4	Acute promyelocytic leukemia	Haematopoietic and lymphoid tissue	135	0.983	0.983
158	NCH82	Glioblastoma	Brain	6	1.000	1.000
159	NCI-H128	Small cell lung cancer	Lung	16	0.909	0.905
160	NCI-H1299	Non-small cell lung carcinoma	Lung	158	0.864	0.853
161	NCI-H187	Small cell lung carcinoma	Lung	74	0.820	0.800
162	NCI-H1975	Bronchoalveolar carcinoma	Lung	259	0.963	0.963
163	NCI-H226	Non-small cell lung carcinoma	Lung	493	0.864	0.860
164	NCI-H23	Non-small cell lung carcinoma	Lung	690	0.862	0.862
165	NCI-H295R	Adrenal cortex carcinoma	Adrenal cortex	11	0.844	0.846
166	NCI-H322M	Non-small cell lung carcinoma	Lung	348	0.852	0.852
167	NCI-H417	Small cell lung carcinoma	Lung	21	0.980	0.981
168	NCI-H46	Lung carcinoma	Lung	6	1.000	1.000
169	NCI-H460	Non-small cell lung carcinoma	Lung	2457	0.901	0.899

170	NCI-H522	Non-small cell lung carcinoma	Lung	614	0.863	0.858
171	NCI-H647	Adenosquamous lung carcinoma	Lung	10	0.887	0.887
172	NCI-H69	Small cell lung carcinoma	Lung	189	0.894	0.894
173	NCI-H838	Non-small cell lung cancer. 3 stage	Lung	19	0.804	0.729
174	NCI-N87	gastric carcinoma	Stomach	228	0.919	0.920
175	NSCLC	Non-small cell lung carcinoma	Lung	64	0.927	0.928
176	NT2	Embryonal carcinoma	Germ cell. fibroblast	58	0.993	0.992
177	NUGC	Gastric carcinoma	Stomach	6	1.000	1.000
178	NUGC-3	Gastric carcinoma	Stomach	228	0.913	0.913
179	OVCAR	Ovarian adenocarcinoma	Ovarium	72	0.975	0.975
180	OVCAR-3	Ovarian adenocarcinoma	Ovarium	1197	0.901	0.901
181	OVCAR-4	Ovarian adenocarcinoma	Ovarium	438	0.830	0.829
182	OVCAR-5	Ovarian adenocarcinoma	Ovarium	375	0.838	0.834
183	OVCAR-8	Ovarian adenocarcinoma	Ovarium	547	0.871	0.867
184	OVXF1023	Ovarian adenocarcinoma	Ovarium	3	1.000	1.000
185	OVXF1353	Ovarian adenocarcinoma	Ovarium	3	1.000	1.000
186	Ovarian carcinoma cells	Ovarian adenocarcinoma	Ovarium	3	0.908	0.910
187	PA-1	Ovarian carcinoma	Ovarium	135	0.841	0.836
188	PANC-1	Pancreatic carcinoma	Pancreas	281	0.919	0.920
189	PAXF546	Pancreatic carcinoma	Pancreas	4	0.807	0.808
190	Panc203	Pancreatic carcinoma	Pancreas	6	1.000	1.000
191	Panc430	Pancreatic carcinoma	Pancreas	8	1.000	1.000
192	PC-3	Prostate carcinoma	Prostate	3410	0.891	0.891
193	PC-6	Small cell lung carcinoma	Lung	5	0.939	0.940
194	PC-9	Lung adenocarcinoma	Lung	10	0.891	0.898
195	PT-45	Pancreatic carcinoma	Pancreas	20	0.933	0.933
196	QG-56	Squamous cell lung carcinoma	Lung	90	1.000	1.000
197	R2	Prostate Carcinoma	Prostate	13	0.996	0.996
198	Raji	B-lymphoblastic	Haematopoietic and lymphoid tissue	155	0.894	0.896
199	Ramos	Burkitts lymhoma B-cells	Blood	41	0.907	0.903
200	RKO	Colon carcinoma	Colon	177	0.835	0.833
201	RPMI 8402	Pre-T-lymphoblastoid cells. acute lymphoblastic leukemia	Haematopoietic and lymphoid tissue	89	0.995	0.995
202	RPMI-8226	Multiple myeloma	Haematopoietic and lymphoid tissue	550	0.858	0.857
203	RT-4	Bladder carcinoma	Urinary tract	40	0.938	0.939
204	RWLeu4	Chronic myelogenous leukemia	Blood	9	1.000	1.000
205	RXF 393	Renal carcinoma	Kidney	429	0.884	0.881
206	RXF 423	Renal carcinoma	Kidney	3	1.000	1.000
207	RXF 944	Renal carcinoma	Kidney	8	0.985	0.985
208	S1B1-20	Colon carcinoma	Colon	38	1.000	1.000
209	SAOS-2	Osteosarcoma	Bone	44	0.933	0.932
210	SF-268	Glioblastoma	Brain	795	0.876	0.870
211	SF-295	Glioblastoma	Brain	629	0.890	0.887
212	SF-539	Glioblastoma	Brain	715	0.906	0.904

213	SGC-7901	Gastric carcinoma	Stomach	214	0.962	0.961
214	SH-SY5Y	Bone marrow neuroblastoma	Brain	197	0.931	0.932
215	SiHa	Cervical squamous cell carcinoma	Cervix	338	0.951	0.949
216	SHP77	Small cell lung carcinoma	Lung	9	0.909	0.911
217	SISO	Uterine cervical adenocarcinoma	Cervix	28	0.949	0.949
218	SJSA-1	Osteosarcoma	Bone	121	0.856	0.849
219	SK-BR-3	Breast adenocarcinoma	Breast	1143	0.954	0.954
220	SK-ES1	Ewing sarcoma	Bone	5	0.809	0.809
221	SK-HEP1	Hepatocellular carcinoma	Liver	101	0.982	0.982
222	SK-MEL	Melanoma	Skin	47	0.966	0.966
223	SK-MEL-1	Metastatic melanoma	Skin	58	0.803	0.809
224	SK-MEL-2	Melanoma	Skin	640	0.871	0.865
225	SK-MEL-28	Melanoma	Skin	786	0.881	0.877
226	SK-MEL-5	Melanoma	Skin	665	0.898	0.898
227	SK-MES-1	Squamous cell lung carcinoma	Lung	126	0.842	0.830
228	SK-N-SH	Neuroblastoma	Nervous system	105	0.924	0.925
229	SK-OV-3	Ovarian carcinoma	Ovarium	1539	0.895	0.893
230	Skut1B	Uterine corpus leiomyosarcoma	Ovarium	4	1.000	1.000
231	SK-VLB	Ovarian carcinoma	Ovarium	17	0.930	0.931
232	SMMC-7721	Hepatocellular carcinoma	Liver	282	0.948	0.942
233	SN12C	Renal carcinoma	Kidney	782	0.883	0.879
234	SNB-7	Glioblastoma	Brain	4	0.998	0.998
235	SNB-75	Glioblastoma	Nervous system	413	0.872	0.872
236	SNU-398	Hepatocellular carcinoma	Liver	73	0.966	0.963
237	SNU-638	Gastric carcinoma	Stomach	94	0.956	0.955
238	SPC-A4	Lung Adenocarcinoma	Lung	10	1.000	1.000
239	SQ20B	Head and neck Squamous carcinoma	Head and neck	33	0.957	0.957
240	SR	Adult immunoblastic lymphoma	Haematopoietic and lymphoid tissue	578	0.864	0.859
241	St-4	Stomach carcinoma	Stomach	30	0.888	0.889
242	ST-KM-1	Gastric carcinoma	Stomach	3	1.000	1.000
243	SW1353	Bone chondrosarcoma	Bone	40	0.956	0.957
244	SW1573	Lung carcinoma	Lung	160	0.901	0.891
245	SW-1736	Thyroid gland undifferentiated (anaplastic) carcinoma	Thyroid	50	0.995	0.995
246	SW480	Colon adenocarcinoma	Colon	524	0.951	0.950
247	SW-60	Colorectal carcinoma	Colon	3	1.000	1.000
248	SW-620	Colon adenocarcinoma	Colon	1223	0.906	0.906
249	T-24	Bladder carcinoma	Urinary tract	187	0.912	0.905
250	T47D	Breast carcinoma	Breast	991	0.885	0.886
251	T98G	Glioblastoma	Brain	129	0.843	0.836
252	TCC-SUP	Bladder Carcinoma	Urinary tract	11	0.941	0.941
253	TF-1	Bone marrow erythroleukemic	Haematopoietic and lymphoid tissue	28	0.987	0.969
254	THP-1	Acute monocytic leukemia	Blood	1105	0.962	0.962
255	TK-10	Renal carcinoma	Kidney	328	0.866	0.867
256	TSGH	Glioma	Brain	40	0.988	0.988

257	TSGH 9201	Gastric carcinoma	Stomach	18	1.000	1.000
258	TSU	Prostatic carcinoma	Prostate	15	0.946	0.946
259	U-251	Glioma	Brain	694	0.875	0.873
260	U-266	Plasma cell myeloma	Blood	108	0.851	0.840
261	U2OS	Osteosarcoma	Bone	80	0.930	0.930
262	U373 MG	Glioblastoma	Brain	154	0.980	0.980
263	U-87 MG	Lymphoblastic lymphoma	Blood	157	0.968	0.968
264	U-937	Histiocytic lymphoma	Haematopoietic and lymphoid tissue	519	0.946	0.946
265	U-937/GTB	Histiocytic lymphoma	Blood	10	0.833	0.834
266	UACC-257	Melanoma	Skin	348	0.835	0.835
267	UACC-375	Melanoma	Skin	86	0.993	0.993
268	UACC-62	Melanoma	Skin	873	0.900	0.901
269	UACC-903	Malignant melanoma	Skin	20	0.999	0.999
270	UCLA P-3	Lung carcinoma cell line	Lung	5	1.000	1.000
271	UMSCC22B	Hypopharyngeal squamous cell carcinoma	Upper aerodigestive tract	4	0.999	0.621
272	UMUC3	Bladder Carcinoma	Urinary tract	21	0.956	0.956
273	UO-31	Renal carcinoma	Kidney	431	0.852	0.847
274	UT7	Leukaemia	Blood	11	1.000	1.000
275	WiDr-NTR	Colon Adenocarcinoma	Colon	8	1.000	1.000
276	XF498	Glioma	Brain	98	0.959	0.959
277	YAPC	Pancreatic carcinoma	Pancreas	49	0.805	0.813
278	ZR-75-1	Breast carcinoma	Breast	204	0.964	0.964
**Mean**					**0.915**	**0.913**

N – number of active compounds in the training set.
