# Supplementary material for: CLC-Pred: A freely available web-service for in silico prediction of human cell line cytotoxicity for drug-like compounds
Source: PLoS One. 2018 Jan 25;13(1):e0191838. doi: 10.1371/journal.pone.0191838 (PMC5784992; doi:10.1371/journal.pone.0191838)
Supplement: S2 Table — (PDF) [file pone.0191838.s003.pdf]

S2 Table.

Pa	Pi	Cell-line	Cell-line name	Tissue/organ
			doxorubicin, cancer cell lines	
0.908	0.001	MES-SA	Uterine corpus sarcoma	Uterus
0.760	0.004	NCI-H838	Non-small cell lung cancer. 3 stage	Lung
0.718	0.002	RXF 944	Renal carcinoma	Kidney
0.680	0.001	MAXF401	Breast carcinoma	Breast
0.661	0.005	DMS-114	Lung carcinoma	Lung
0.653	0.000	UCLA P-3	Lung carcinoma cell line	Lung
0.629	0.000	KBM-3	Acute myelomonocytic leukemia	Blood
0.629	0.000	KBM-3/DOX	Acute myelomonocytic leukemia	Blood
0.609	0.004	NCI-H187	Small cell lung carcinoma	Lung
0.620	0.029	MCF7	Breast carcinoma	Breast
0.595	0.011	SF-268	Glioblastoma	Brain
0.586	0.004	A2058	Melanoma	Skin
0.580	0.001	LXFL 529	Non-small cell lung carcinoma	Lung
0.569	0.000	SK-ES1	Ewing sarcoma	Bone
0.580	0.015	HepG2	Hepatoblastoma	Liver
0.564	0.004	MDA-MB-453	Breast adenocarcinoma	Breast
0.549	0.000	SHP77	Small cell lung carcinoma	Lung
0.551	0.004	SK-MES-1	Squamous cell lung carcinoma	Lung
0.558	0.017	UACC-257	Melanoma	Skin
0.529	0.003	MES-SA/DXS	Uterine corpus sarcoma	Uterus
0.523	0.004	YAPC	Pancreatic carcinoma	Pancreas
0.532	0.018	NCI-H322M	Non-small cell lung carcinoma	Lung
0.528	0.016	TK-10	Renal carcinoma	Kidney
0.526	0.019	OVCAR-5	Ovarian adenocarcinoma	Ovarium
0.508	0.008	U-266	Plasma cell myeloma	Blood
0.512	0.019	A498	Renal carcinoma	Kidney
0.493	0.001	L2987	Lung adenocarcinoma	Lung
0.496	0.017	SK-MEL-28	Melanoma	Skin
0.491	0.013	RPMI-8226	Multiple myeloma	Haematopoietic and lymphoid tissue
0.480	0.006	HOS	Osteosarcoma	Bone
0.498	0.027	HL-60	Promyeloblast leukemia	Haematopoietic and lymphoid tissue
0.487	0.023	OVCAR-4	Ovarian adenocarcinoma	Ovarium
0.462	0.011	RKO	Colon carcinoma	Colon
0.451	0.004	A2780cisR	Cisplatin-resistant ovarian carcinoma	Ovarium
0.447	0.007	NALM-6	Adult B acute lymphoblastic leukemia	Haematopoietic and lymphoid tissue
0.445	0.005	T-24	Bladder carcinoma	Urinary tract
0.445	0.006	Caco-2	Colon adenocarcinoma	Colon
0.434	0.001	Human lung LXF 629L tumor	Lung adenocarcinoma	Lung
0.453	0.024	HOP-92	Non-small cell lung carcinoma	Lung
0.443	0.022	BT-549	Breast ductal carcinoma	Breast
0.426	0.005	SJSA-1	Osteosarcoma	Bone
0.427	0.012	PA-1	Ovarian carcinoma	Ovarium
0.440	0.025	SNB-75	Glioblastoma	Nervous system
0.435	0.028	Malme-3M	Melanoma	Skin
0.413	0.007	HuP-T3	Pancreatic adenocarcinoma	Pancreas
0.430	0.031	OVCAR-8	Ovarian adenocarcinoma	Ovarium
0.432	0.033	NCI-H226	Non-small cell lung carcinoma	Lung
0.404	0.009	T98G	Glioblastoma	Brain
0.419	0.030	UO-31	Renal carcinoma	Kidney
0.390	0.002	U-937/GTB	Histiocytic lymphoma	Blood
0.372	0.002	HOS-TE85	Osteosarcoma	Bone
0.369	0.004	MDA-MB-361	Breast adenocarcinoma	Breast
0.397	0.037	SK-MEL-2	Melanoma	Skin

0.364	0.008	CWR22R	Prostate carcinoma epithelial cell line	Prostate
0.380	0.025	RXF 393	Renal carcinoma	Kidney
0.363	0.011	Raji	B-lymphoblastic cells	Haematopoietic and lymphoid tissue
0.398	0.046	MOLT-4	Acute T-lymphoblastic leukemia	Blood
0.357	0.008	C8166	Leukemic T-cells	Blood
0.363	0.015	AGS	Gastric adenocarcinoma	Stomach
0.353	0.006	G-361	Melanoma	Skin
0.382	0.035	OVCAR-3	Ovarian adenocarcinoma	Ovarium
0.380	0.043	SN12C	Renal carcinoma	Kidney
0.418	0.083	SK-MEL-1	Metastatic melanoma	Skin
0.346	0.013	5637	Urothelial bladder carcinoma	Urinary tract
0.380	0.046	DU-145	Prostate carcinoma	Prostate
0.333	0.009	NCI-H69	Small cell lung carcinoma	Lung
0.351	0.027	8505C	Thyroid gland undifferentiated (anaplastic) carcinoma	Thyroid
0.365	0.041	SR	Adult immunoblastic lymphoma	Haematopoietic and lymphoid tissue
0.360	0.037	SF-539	Glioblastoma	Brain
0.331	0.009	KARPAS-299	Anaplastic large cell lymphoma	Haematopoietic and lymphoid tissue
0.321	0.000	H2981	Lung carcinoma	Lung
0.318	0.004	SK-MEL	Melanoma	Skin
0.369	0.057	K562	Erythroleukemia	Haematopoietic and lymphoid tissue
0.324	0.017	HT-1080	Fibrosarcoma	Soft tissue
0.307	0.010	BXPC-3	Pancreatic adenocarcinoma	Pancreas
0.351	0.056	CAKI-1	Kidney carcinoma	Kidney
0.339	0.052	NCI-H522	Non-small cell lung carcinoma	Lung
0.305	0.018	A-427	Lung carcinoma	Lung
0.313	0.026	NCI-H1299	Non-small cell lung carcinoma	Lung
0.322	0.045	UACC-62	Melanoma	Skin
0.309	0.032	PANC-1	Pancreatic carcinoma	Pancreas
0.283	0.007	SW1573	Lung carcinoma	Lung
0.313	0.047	SF-295	Glioblastoma	Brain
0.341	0.076	NCI-H23	Non-small cell lung carcinoma	Lung
0.281	0.017	BGC-823	Stomach adenocarcinoma	Stomach
0.319	0.057	IGROV-1	Ovarian adenocarcinoma	Ovarium
0.325	0.073	SK-OV-3	Ovarian carcinoma	Ovarium
0.254	0.003	SK-HEP1	Hepatocellular carcinoma	Liver
0.317	0.070	KM12	Colon adenocarcinoma	Colon
0.258	0.013	SH-SY5Y	Bone marrow neuroblastoma	Brain
0.259	0.015	J82	Bladder carcinoma	Urinary tract
0.247	0.007	U2OS	Osteosarcoma	Bone
0.237	0.001	UMUC3	Bladder Carcinoma	Urinary tract
0.292	0.063	HOP-62	Non-small cell lung carcinoma	Lung
0.294	0.065	ACHN	Papillary renal carcinoma	Kidney
0.230	0.009	CAOV-3	High grade ovarian serous adenocarcinoma	Ovarium
0.291	0.070	U-251	Glioma	Brain
0.265	0.048	Bel-7402	Hepatoma	Liver
0.221	0.005	IMR-32	Neuroblastoma	Nervous system
0.220	0.005	SNU-638	Gastric carcinoma	Stomach
0.280	0.067	SK-MEL-5	Melanoma	Skin
0.213	0.004	MOLT-3	T-lymphoblastic leukemia	Blood
0.203	0.003	NCI-H417	Small cell lung carcinoma	Lung
0.194	0.003	Bcap37	Breast adenocarcinoma	Breast
0.195	0.006	SK-VLB	Ovarian carcinoma	Ovarium
0.217	0.031	U-937	Histiocytic lymphoma	Haematopoietic and lymphoid tissue
0.249	0.064	LOX IMVI	Melanoma	Skin
0.188	0.007	SK-N-SH	Neuroblastoma	Nervous system
0.187	0.007	SNU-398	Hepatocellular carcinoma	Liver
0.208	0.034	NUGC-3	Gastric carcinoma	Stomach
0.255	0.084	MDA-MB-468	Breast adenocarcinoma	Breast

0.215	0.046	Jurkat	Acute leukemic T-cells	Blood
0.251	0.083	CCRF-CEM	Childhood T acute lymphoblastic leukemia	Blood
0.198	0.031	NCI-N87	gastric carcinoma	Stomach
0.165	0.004	Col2	Colon carcinoma	Colon
0.191	0.031	SMMC-7721	Hepatocellular carcinoma	Liver
0.204	0.044	A-375	Malignant melanoma	Skin
0.175	0.017	SGC-7901	Gastric carcinoma	Stomach
0.252	0.095	M14	Melanoma	Skin
0.166	0.009	ASPC1	Pancreatic ductal adenocarcinoma	Pancreas
0.201	0.045	DLD-1	Colon adenocarcinoma	Colon
0.223	0.070	MIA PaCa-2	Pancreatic carcinoma	Pancreas
0.161	0.009	XF498	Glioma	Brain
0.239	0.100	NCI-H460	Non-small cell lung carcinoma	Lung
0.150	0.013	NSCLC	Non-small cell lung carcinoma	Lung
0.128	0.004	CCRF-SB	Childhood T acute lymphoblastic leukemia	Blood
0.136	0.016	CH1	Ovarian carcinoma	Ovarium
0.134	0.024	ZR-75-1	Breast carcinoma	Breast
0.239	0.130	786-0	Renal carcinoma	Kidney
0.111	0.008	U373 MG	Glioblastoma	Brain
0.157	0.078	ADR5000	Childhood T acute lymphoblastic leukemia	Blood
0.081	0.009	OVCAR	Ovarian adenocarcinoma	Ovarium
0.084	0.013	U-87 MG	Lymphoblastic lymphoma	Blood
0.114	0.045	HCT-8	Ileocecal adenocarcinoma	Large intestine
0.207	0.150	T47D	Breast carcinoma	Breast
0.201	0.161	MDA-MB-231	Breast adenocarcinoma	Breast
0.277	0.242	CFPAC-1	Pancreatic carcinoma	Pancreas
0.102	0.073	SW480	Colon adenocarcinoma	Colon
0.117	0.090	LNCaP	Prostate carcinoma	Prostate
0.135	0.110	A2780	Ovarian carcinoma	Ovarium
0.092	0.070	SiHa	Cervical squamous cell carcinoma	Cervix
0.078	0.061	KETR3	Renal carcinoma	Kidney
0.178	0.168	HCT-15	Colon adenocarcinoma	Colon
0.020	0.018	UACC-375	Melanoma	Skin
doxorubicin, normal cell lines				
0.960	0.002	WI-38 VA13	Embryonic lung fibroblast	Lung
0.822	0.000	HEL 299	Fibroblast	Lung
0.804	0.002	HMEC	Microvascular endothelial cell	Breast
0.625	0.008	MRC5	Embryonic lung fibroblast	Lung
0.558	0.004	BJ	Foreskin fibroblast	Foreskin
0.547	0.001	NHDF	Fibroblast	Skin
0.532	0.000	IMR-90	Embryonic lung fibroblast	Lung
0.402	0.001	CRL-7065	Fibroblast	Skin
0.373	0.002	WIL2-NS	Lymphoblastoid cell	Haematopoietic, lymphoid tissue
0.249	0.003	Detroit 551	Embryonic skin	Skin
0.205	0.009	HaCaT	Keratinocyte	Skin
0.259	0.075	HEK293	Embryonic kidney fibroblast	Kidney
0.098	0.090	WI-38	Embryonic lung fibroblast	Lung
gemcitabine, cancer cell lines				
0.821	0.003	NCI-H838	Non-small cell lung cancer. 3 stage	Lung
0.818	0.001	HPAC	Pancreatic adenocarcinoma	Pancreas
0.804	0.001	CFPAC-1	Pancreatic carcinoma	Pancreas
0.764	0.006	OVCAR-5	Ovarian adenocarcinoma	Ovarium
0.757	0.005	BT-549	Breast ductal carcinoma	Breast
0.750	0.007	HOP-92	Non-small cell lung carcinoma	Lung
0.733	0.002	PC-9	Lung adenocarcinoma	Lung
0.730	0.003	Caco-2	Colon adenocarcinoma	Colon
0.720	0.003	A2058	Melanoma	Skin
0.716	0.001	COLO 320	Colon adenocarcinoma	Colon

0.689	0.006	UACC-257	Melanoma	Skin
0.687	0.006	HeLa	Cervical adenocarcinoma	Cervix
0.674	0.009	OVCAR-4	Ovarian adenocarcinoma	Ovarium
0.669	0.005	NCI-H23	Non-small cell lung carcinoma	Lung
0.666	0.009	NCI-H226	Non-small cell lung carcinoma	Lung
0.647	0.008	UO-31	Renal carcinoma	Kidney
0.611	0.004	NCI-H187	Small cell lung carcinoma	Lung
0.609	0.010	SF-268	Glioblastoma	Brain
0.611	0.012	786-0	Renal carcinoma	Kidney
0.601	0.010	OVCAR-8	Ovarian adenocarcinoma	Ovarium
0.594	0.009	CCRF-CEM	Childhood T acute lymphoblastic leukemia	Blood
0.575	0.004	T-24	Bladder carcinoma	Urinary tract
0.573	0.003	HuP-T3	Pancreatic adenocarcinoma	Pancreas
0.577	0.013	Kasumi 1	Childhood acute myeloid leukemia with maturation	Haematopoietic and lymphoid tissue
0.566	0.004	PA-1	Ovarian carcinoma	Ovarium
0.565	0.004	HOS	Osteosarcoma	Bone
0.567	0.012	EKVX	Non-small cell lung carcinoma	Lung
0.555	0.005	U-266	Plasma cell myeloma	Blood
0.551	0.004	SK-MES-1	Squamous cell lung carcinoma	Lung
0.565	0.017	MOLT-4	Acute T-lymphoblastic leukemia	Blood
0.553	0.014	SK-MEL-2	Melanoma	Skin
0.541	0.005	RKO	Colon carcinoma	Colon
0.548	0.015	TK-10	Renal carcinoma	Kidney
0.549	0.016	NCI-H322M	Non-small cell lung carcinoma	Lung
0.530	0.001	CEM-SS	Childhood T acute lymphoblastic leukemia	Blood
0.539	0.013	M14	Melanoma	Skin
0.537	0.014	U-251	Glioma	Brain
0.509	0.001	SNB-7	Glioblastoma	Brain
0.509	0.018	IGROV-1	Ovarian adenocarcinoma	Ovarium
0.495	0.004	Raji	B-lymphoblastic cells	Haematopoietic and lymphoid tissue
0.464	0.002	C8166	Leukemic T-cells	Blood
0.462	0.004	SJSA-1	Osteosarcoma	Bone
0.462	0.005	NALM-6	Adult B acute lymphoblastic leukemia	Haematopoietic and lymphoid tissue
0.457	0.004	T98G	Glioblastoma	Brain
0.471	0.019	YAPC	Pancreatic carcinoma	Pancreas
0.452	0.006	HT-1080	Fibrosarcoma	Soft tissue
0.444	0.006	AGS	Gastric adenocarcinoma	Stomach
0.442	0.004	CWR22R	Prostate carcinoma epithelial cell line	Prostate
0.438	0.003	SW1573	Lung carcinoma	Lung
0.433	0.003	MES-SA/DXS	Uterine corpus sarcoma	Uterus
0.430	0.005	A-427	Lung carcinoma	Lung
0.470	0.044	SK-MEL-1	Metastatic melanoma	Skin
0.443	0.020	ACHN	Papillary renal carcinoma	Kidney
0.426	0.013	H9	T-lymphoid	Haematopoietic and lymphoid tissue
0.412	0.000	ST-KM-1	Gastric carcinoma	Stomach
0.444	0.033	MDA-MB-453	Breast adenocarcinoma	Breast
0.435	0.029	A498	Renal carcinoma	Kidney
0.407	0.008	PANC-1	Pancreatic carcinoma	Pancreas
0.459	0.062	DMS-114	Lung carcinoma	Lung
0.397	0.004	5637	Urothelial bladder carcinoma	Urinary tract
0.432	0.040	HL-60	Promyeloblast leukemia	Haematopoietic and lymphoid tissue
0.412	0.031	SN12C	Renal carcinoma	Kidney
0.407	0.034	KM12	Colon adenocarcinoma	Colon
0.378	0.010	NCI-H1299	Non-small cell lung carcinoma	Lung
0.385	0.018	8505C	Thyroid gland undifferentiated (anaplastic) carcinoma	Thyroid
0.385	0.027	UACC-62	Melanoma	Skin
0.387	0.030	SF-539	Glioblastoma	Brain
0.356	0.008	BXPC-3	Pancreatic adenocarcinoma	Pancreas

0.375	0.036	NCI-H522	Non-small cell lung carcinoma	Lung
0.344	0.005	KARPAS-299	Anaplastic large cell lymphoma	Haematopoietic and lymphoid tissue
0.344	0.006	MDA-MB-361	Breast adenocarcinoma	Breast
0.345	0.007	G-361	Melanoma	Skin
0.336	0.002	COLO 320DM	Colon adenocarcinoma	Colon
0.330	0.005	J82	Bladder carcinoma	Urinary tract
0.352	0.030	COLO 205	Colon adenocarcinoma	Colon
0.360	0.042	RPMI-8226	Multiple myeloma	Haematopoietic and lymphoid tissue
0.355	0.042	OVCAR-3	Ovarian adenocarcinoma	Ovarium
0.347	0.049	SR	Adult immunoblastic lymphoma	Haematopoietic and lymphoid tissue
0.309	0.015	NCI-H69	Small cell lung carcinoma	Lung
0.295	0.005	U2OS	Osteosarcoma	Bone
0.306	0.035	MIA PaCa-2	Pancreatic carcinoma	Pancreas
0.277	0.008	NUGC-3	Gastric carcinoma	Stomach
0.333	0.065	CAKI-1	Kidney carcinoma	Kidney
0.316	0.055	HOP-62	Non-small cell lung carcinoma	Lung
0.242	0.008	CAOV-3	High grade ovarian serous adenocarcinoma	Ovarium
0.305	0.071	HCT-15	Colon adenocarcinoma	Colon
0.279	0.049	LOX IMVI	Melanoma	Skin
0.222	0.004	U373 MG	Glioblastoma	Brain
0.223	0.011	NCI-N87	gastric carcinoma	Stomach
0.269	0.073	SK-MEL-5	Melanoma	Skin
0.187	0.002	KKLS	Gastric adenocarcinoma	Stomach
0.190	0.005	MT4	Adult T acute lymphoblastic leukemia	Blood
0.261	0.080	MDA-MB-468	Breast adenocarcinoma	Breast
0.264	0.095	T47D	Breast carcinoma	Breast
0.215	0.051	A-431	Epidermoid carcinoma	Skin
0.254	0.107	K562	Erythroleukemia	Haematopoietic and lymphoid tissue
0.163	0.017	SNU-638	Gastric carcinoma	Stomach
0.136	0.004	NCI-H417	Small cell lung carcinoma	Lung
0.121	0.006	KATO III stomach cancer	Signet ring cell gastric adenocarcinoma	Stomach
0.145	0.043	LNCaP	Prostate carcinoma	Prostate
0.105	0.004	CCRF-HSB-2	Childhood T acute lymphoblastic leukemia	Blood
0.144	0.044	SW480	Colon adenocarcinoma	Colon
0.191	0.092	Bel-7402	Hepatoma	Liver
0.256	0.172	MCF7	Breast carcinoma	Breast
0.220	0.149	Malme-3M	Melanoma	Skin
0.220	0.153	SK-MEL-28	Melanoma	Skin
0.109	0.046	MKN-45	Gastric adenocarcinoma	Stomach
0.083	0.036	TSU	Prostatic carcinoma	Prostate
0.046	0.005	CEM-c113	Childhood T acute lymphoblastic leukemia	Blood
0.171	0.133	St-4	Stomach carcinoma	Stomach
0.187	0.151	SF-295	Glioblastoma	Brain
0.191	0.156	SK-OV-3	Ovarian carcinoma	Ovarium
0.106	0.085	Huh-7	Hepatocellular carcinoma	Liver
0.216	0.201	A549	Lung carcinoma	Lung
0.133	0.120	LoVo	Colon adenocarcinoma	Colon
0.116	0.106	DLD-1	Colon adenocarcinoma	Colon
gemcitabine, normal cell lines				
0.528	0.004	BJ	Foreskin fibroblast	Foreskin
0.362	0.004	NHDF	Fibroblast	Skin
0.321	0.021	HEL 299	Fibroblast	Lung
0.169	0.039	HUVEC	Umbilical vein endothelial cell	Endothelium
0.163	0.039	HFF	Foreskin fibroblast	Skin
raloxifene, cancer cell lines				
0.865	0.006	MCF7	Breast carcinoma	Breast
0.336	0.065	MOLT-4	Acute T-lymphoblastic leukemia	Blood

0.323	0.005	U2OS	Osteosarcoma	Bone
0.294	0.074	HeLa	Cervical adenocarcinoma	Cervix
0.227	0.157	HT-29	Colon adenocarcinoma	Colon
0.227	0.196	PC-9	Lung adenocarcinoma	Lung
0.225	0.130	K562	Erythroleukemia	Haematopoietic and lymphoid tissue
0.217	0.198	Hs-578T	Invasive ductal breast carcinoma	Breast
0.208	0.116	CCRF-CEM	Childhood T acute lymphoblastic leukemia	Blood
0.169	0.004	Ishikawa	Endometrial adenocarcinoma	Uterus
0.161	0.110	Ramos	Burkitts lymhoma B-cells	Blood
0.157	0.122	Caco-2	Colon adenocarcinoma	Colon
0.156	0.043	MES-SA/DXS	Uterine corpus sarcoma	Uterus
0.153	0.046	HOS-TE85	Osteosarcoma	Bone
0.145	0.028	MES-SA	Uterine corpus sarcoma	Uterus
0.107	0.038	SQ20B	Head and neck Squamous carcinoma	Head and neck
0.095	0.083	MKN-45	Gastric adenocarcinoma	Stomach
0.064	0.062	MCF7S	Breast carcinoma	Breast
0.045	0.022	HA22T	Hepatocellular carcinoma	Liver
0.039	0.015	CEM-0	T-cell leukemia	Blood
0.031	0.014	CCRF-CEM/VCR-1000	T-cell leukaemia	Blood
0.024	0.019	GC3/Cl	Colorectal carcinoma	Colon
0.021	0.017	HRT-18	Colon adenocarcinoma	Colon
raloxifene, normal cell lines				
0.104	0.046	Detroit 551	Embryonic skin	Skin
0.087	0.065	CRL-7065	Fibroblast	Skin
vinorelbine, cancer cell lines				
0.931	0.005	A549	Lung carcinoma	Lung
0.896	0.005	HCT-116	Colon carcinoma	Colon
0.842	0.004	PC-6	Small cell lung carcinoma	Lung
0.802	0.005	HeLa	Cervical adenocarcinoma	Cervix
0.689	0.008	NCI-H838	Non-small cell lung cancer. 3 stage	Lung
0.635	0.007	DMS-114	Lung carcinoma	Lung
0.619	0.005	Kasumi 1	Childhood acute myeloid leukemia with maturation	Haematopoietic and lymphoid tissue
0.597	0.005	SK-MEL-1	Metastatic melanoma	Skin
0.580	0.004	NCI-H295R	Adrenal cortex carcinoma	Adrenal cortex
0.544	0.004	PA-1	Ovarian carcinoma	Ovarium
0.526	0.005	MDA-MB-453	Breast adenocarcinoma	Breast
0.519	0.005	YAPC	Pancreatic carcinoma	Pancreas
0.518	0.009	A2058	Melanoma	Skin
0.501	0.014	NCI-H187	Small cell lung carcinoma	Lung
0.490	0.009	U-266	Plasma cell myeloma	Blood
0.452	0.021	CFPAC-1	Pancreatic carcinoma	Pancreas
0.425	0.005	SJSA-1	Osteosarcoma	Bone
0.420	0.000	HCT-116/VM46	Colon carcinoma	Colon
0.420	0.013	SK-MES-1	Squamous cell lung carcinoma	Lung
0.413	0.008	T98G	Glioblastoma	Brain
0.412	0.022	RKO	Colon carcinoma	Colon
0.406	0.026	NALM-6	Adult B acute lymphoblastic leukemia	Haematopoietic and lymphoid tissue
0.401	0.008	HuP-T3	Pancreatic adenocarcinoma	Pancreas
0.371	0.019	HOS	Osteosarcoma	Bone
0.356	0.025	8505C	Thyroid gland undifferentiated (anaplastic) carcinoma	Thyroid
0.347	0.012	C8166	Leukemic T-cells	Blood
0.343	0.013	CWR22R	Prostate carcinoma epithelial cell line	Prostate
0.340	0.005	KARPAS-299	Anaplastic large cell lymphoma	Haematopoietic and lymphoid tissue
0.329	0.025	5637	Urothelial bladder carcinoma	Urinary tract
0.319	0.086	OVCAR-5	Ovarian adenocarcinoma	Ovarium
0.313	0.031	AGS	Gastric adenocarcinoma	Stomach
0.300	0.034	NCI-H1299	Non-small cell lung carcinoma	Lung

0.297	0.119	UACC-257	Melanoma	Skin
0.296	0.024	NCI-H69	Small cell lung carcinoma	Lung
0.293	0.025	A-427	Lung carcinoma	Lung
0.283	0.086	H9	T-lymphoid	Haematopoietic and lymphoid tissue
0.279	0.031	MDA-MB-361	Breast adenocarcinoma	Breast
0.274	0.034	G-361	Melanoma	Skin
0.266	0.026	T-24	Bladder carcinoma	Urinary tract
0.261	0.177	OVCAR-4	Ovarian adenocarcinoma	Ovarium
0.260	0.046	Raji	B-lymphoblastic cells	Haematopoietic and lymphoid tissue
0.252	0.004	PT-45	Pancreatic carcinoma	Pancreas
0.244	0.025	J82	Bladder carcinoma	Urinary tract
0.243	0.174	HOP-92	Non-small cell lung carcinoma	Lung
0.242	0.141	UO-31	Renal carcinoma	Kidney
0.230	0.012	ZR-75-1	Breast carcinoma	Breast
0.225	0.161	NCI-H226	Non-small cell lung carcinoma	Lung
0.224	0.063	HT-1080	Fibrosarcoma	Soft tissue
0.220	0.017	SW1573	Lung carcinoma	Lung
0.217	0.021	SMMC-7721	Hepatocellular carcinoma	Liver
0.210	0.011	CAOV-3	High grade ovarian serous adenocarcinoma	Ovarium
0.193	0.046	NUGC-3	Gastric carcinoma	Stomach
0.183	0.105	MIA PaCa-2	Pancreatic carcinoma	Pancreas
0.170	0.028	MES-SA/DXS	Uterine corpus sarcoma	Uterus
0.169	0.090	NCI-N87	gastric carcinoma	Stomach
0.124	0.018	SNU-398	Hepatocellular carcinoma	Liver
0.109	0.074	Huh-7	Hepatocellular carcinoma	Liver
0.097	0.053	SiHa	Cervical squamous cell carcinoma	Cervix
vinorelbine, normal cell lines				
0.993	0.003	HUVEC	Umbilical vein endothelial cell	Endothelium
0.313	0.045	MRC5	Embryonic lung fibroblast	Lung
